# Three Dimensional Glomerular Reconstruction: A Novel Approach to Evaluate Renal Microanatomy in Diabetic Kidney Disease

**DOI:** 10.1038/s41598-019-38646-z

**Published:** 2019-02-12

**Authors:** Niloufar Torkamani, George Jerums, Paul Crammer, Alison Skene, David A. Power, Sianna Panagiotopoulos, Michele Clarke, Richard J. MacIsaac, Elif I. Ekinci

**Affiliations:** 1 Department of Medicine, University of Melbourne, Austin Health, Heidelberg, Victoria Australia; 2 Department of Endocrinology, Austin Health, Melbourne, Victoria Australia; 3 Department of Anatomical Pathology, Monash Medical Centre, Melbourne, Victoria Australia; 4 Department of Nephrology, Austin Health, Melbourne, Victoria Australia; 5 Department of Endocrinology & Diabetes, St. Vincent’s Hospital Melbourne and University of Melbourne, Fitzroy, Victoria Australia

## Abstract

Mesangial metrics reflect glomerular filtration surface area in diabetes. The point-sampled intercept (PSI) method is the conventional method to calculate these parameters. However, this is time consuming and subject to underestimation. We introduce a novel three-dimensional (3D) reconstruction method applicable to light microscopy to measure mesangial metrics. Transmission electron microscopy (TEM), PSI and our new 3D imaging methods were used to quantify mesangial metrics from 22 patients with type 2 diabetes, normo-, micro- and macroalbuminuria and an estimated glomerular filtration rate of <60 mL/min/1.73 m^2^. Repeated-measures ANOVA test was used to test the equality of the measurement means from the three methods and the degree of inter method variability. Repeated-measures and post-estimation ANOVA tests together with correlation coefficient measurements were used to compare the methods with TEM as reference. There was a statistically significant difference in mesangial volume measurements (*F*(2, 16) = 15.53, *p* = 0.0002). The PSI method underestimated measurements compared to TEM and 3D methods by 30% (*p* = 0.001) and 15%, respectively (*p* < 0.001). 3D and TEM measurements did not differ significantly. 3D reconstruction is a reliable and time efficient method for calculating mesangial metrics. It may prove to be a useful tool in clinical and experimental diabetic kidney disease.

## Introduction

Diabetes is the leading cause of chronic kidney disease (CKD) worldwide^1^. It is therefore important to understand the microstructural changes caused by diabetes that result in nephropathy. A better understanding of the structural changes associated with CKD can lead to the identification of novel risk factors and promoters of diabetic kidney disease (DKD).

While qualitative changes in the renal structure in DKD have been widely described^2^, quantitative studies of structural-functional relationships in human DKD are rare^3^. Conventional methods to quantify renal structure in diabetes are highly dependent on electron microscopy and the point sample intercept (PSI) method^3^ which are expensive, time consuming and require a high degree of technical skill to achieve optimal results. The PSI method, which is based on Cavalieri’s method of segmentation, has demonstrated underestimation of cell volume and surface area^4^. We hypothesised that a novel technique using computer assisted three-dimensional (3D) imaging can be applied to LM sections to measure mesangial metrics. The primary outcome of the study was therefore to assess the applicability of a 3D method in measurement of mesangial volumes compared to PSI and TEM methods. An additional outcome was to determine if there was an association between the degree of albuminuria and mesangial volumes.

## Results

Three dimensional reconstructions were generated for 63 glomeruli from 22 patients. All three methods of calculating the mesangial volume using 3D, PSI and TEM were used in 18 glomeruli from 8 patients.

The baseline characteristics of the study participants are shown in Table 1. There was a trend for a higher proportion of women to be in the normoalbuminuria group (p < 0.05). There were more smokers (p < 0.05) in the microalbuminuria group. The estimated glomerular filtration rate (eGFR) calculated using the Chronic Kidney Disease Epidemiology Collaboration (CKD-EPI) equation was lower in the macroalbuminuria group (p < 0.005). Otherwise there were no statistical differences between the groups.

**Table 1:** Baseline Characteristics.

Characteristic (n = 22)	Normoalbuminuria (n = 8)	Microalbuminuria (n = 6)	Macroalbuminuria (n = 8)	P value
Gender	3 M, 5 F	5 M, 1 F	8 M	0.04
Age (years)	67 ± 2*	69 ± 2.8	64 ± 6.8	0.16
Duration (years)	12 ± 2.4	12 ± 3.5	18.5 ± 8	0.17
BMI (kg/m^2^)	34 ± 1.6	33 ± 2.7	29.5 ± 4.6	0.51
AER (µg/min)	7.9 ± 1.2	113 ± 19	2,276 ± 1,818	
eGFR (ml/min/1.73 m^2^)	41 ± 3	48 ± 4	37 ± 11	0.003
Retinopathy	4/8	3/6	8/8	0.21
Smoker	0/8	3/6	2/8	0.03
HbA1c (%)	6.8 ± 0.2	8 ± 0.5	8.4 ± 1.4	0.31
Total Cholesterol (mmol/L)	4.4 ± 0.2	4.1 ± 0.4	4.5 ± 1.4	0.5

^*^Data shown as mean ± SD. P values were determined by Fisher’s exact test for categorical variables and Kruskal-Wallis for continuous variables. F, female; M, male.

**Figure 1:**
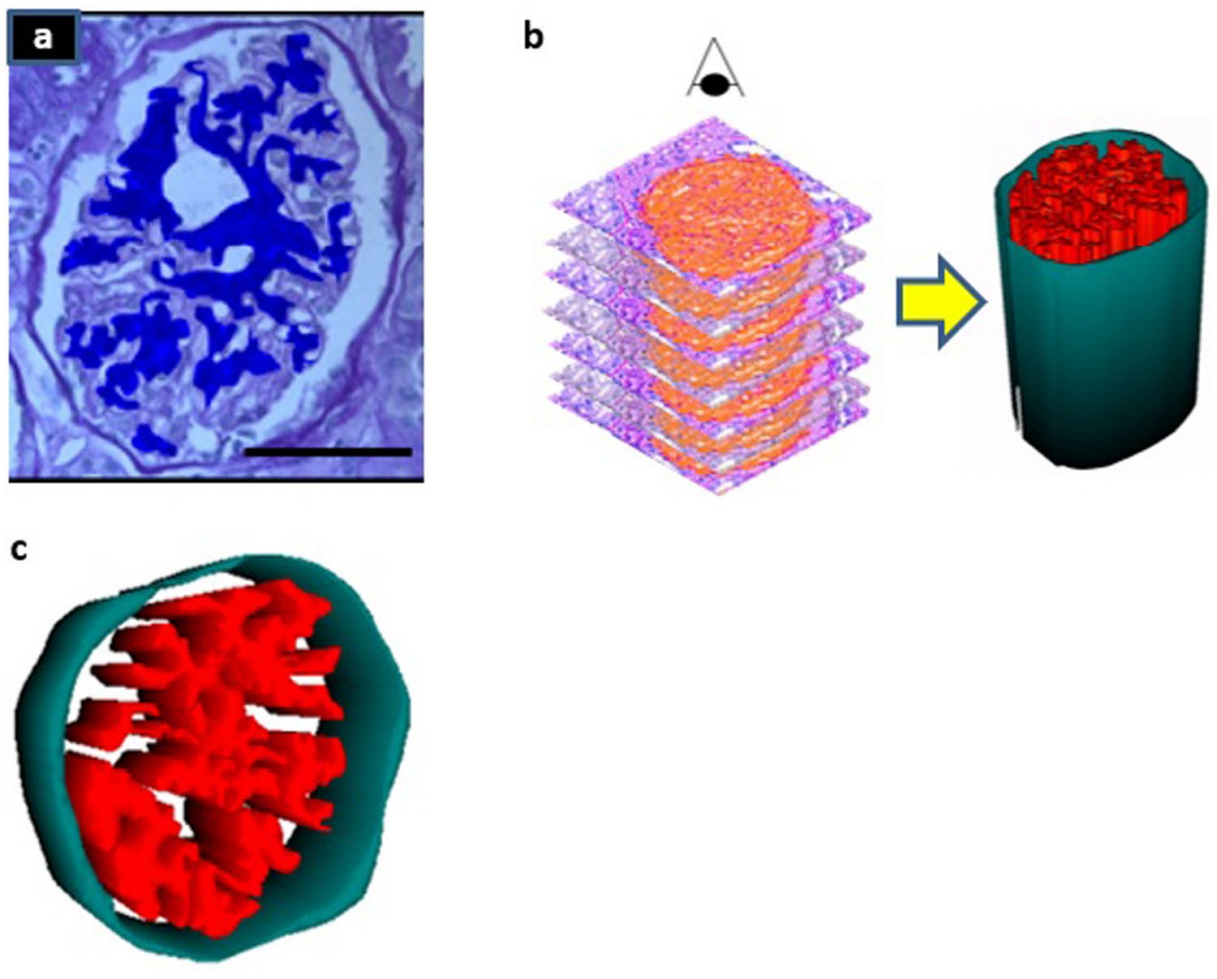
Three-dimensional reconstruction of the glomerulus. (**a**) Glomerular Mesangium (blue) was identified using Reconstruct software based on PAS staining differentiation (scale bar = 50 μm). The eye symbol represents the direction of the view (**b**) Superior view of glomerulus. 3-dimensional model of the glomerulus was generated using serial horizontal (transverse) sections (**c**) Close-up view of the 3D model of the glomerular mesangial area. Glomerular mesangial (red) surface area and volume was quantified.

To validate the results from the 3D reconstruction method, mesangial volumes were measured by the 3D method and by using the TEM and PSI methods. Figure 1a shows a PAS stained histological section of the glomerulus where glomerular mesangium (blue) was identified using “Reconstruct software” based on PAS differential staining. An example of three-dimensional reconstruction of the glomerulus is shown in Fig. 1b,c. Figure 1b demonstrates a 3D model of the glomerulus expanded in the z direction (superior view) resulting from reconstruction of multiple horizontal transverse serial sections. Glomerular mesangium is represented in red and the surface area and volume of mesangium were quantified for each glomerulus. As the reconstruction is limited by the number of serial sections the shape resembles a cylinder. As the number of sections is increased, this will gradually convert to a shape that is closer to the real-life glomerular shape.

**Figure 2:**
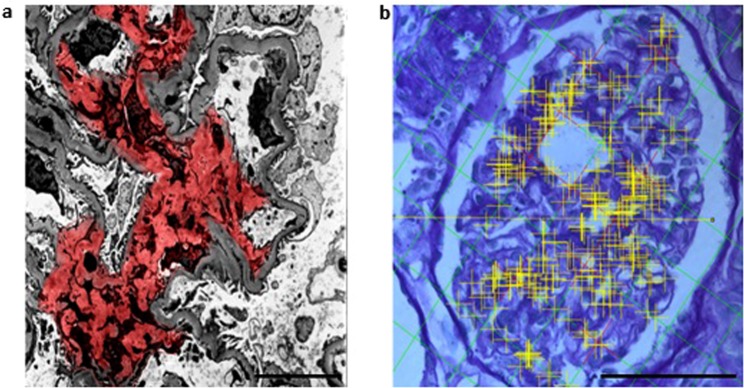
Mesangial area quantification using Transmission Electron Microscopy (TEM) and Point Sample Intercept (PSI) methods. (**a**) Mesangial area and estimated volume was calculated from TEM images (Scale bar 4 µm). Images were analysed (Digimizer 4.2.2) and mesangial area (red) was identified by an expert operator. Transverse sections through the middle of representative glomeruli were chosen and all identifiable mesangial area per glomerulus was measured. The mean mesangial area in a glomerulus per subject was then calculated. (**b**) Fractional volumes of glomerular components were calculated using PSI method. Using the Cavalieri’s method of segmentation combined with automated point counting (yellow) the mesangial volume was estimated (Fiji by Image J 1.51e) (Scale bar 50 µm).

Figure 2 shows representative sections of the glomerulus that were used to estimate mesangial volumes using the TEM and PSI methods. Mesangial area and estimated volume was calculated from TEM images. These images were analysed (Digimizer 4.2.2) and mesangial area (red) was identified by an expert operator. Transverse sections through the middle of representative glomeruli were chosen and all identifiable mesangial area per glomerulus was measured. The mean mesangial area in a glomerulus per subject was then calculated. Fractional volumes of glomerular components were also calculated using PSI method. Using the Cavalieri’s method of segmentation combined with automated point counting (yellow) the mesangial volume was estimated (Fiji by Image J 1.51e).

**Figure 3:**
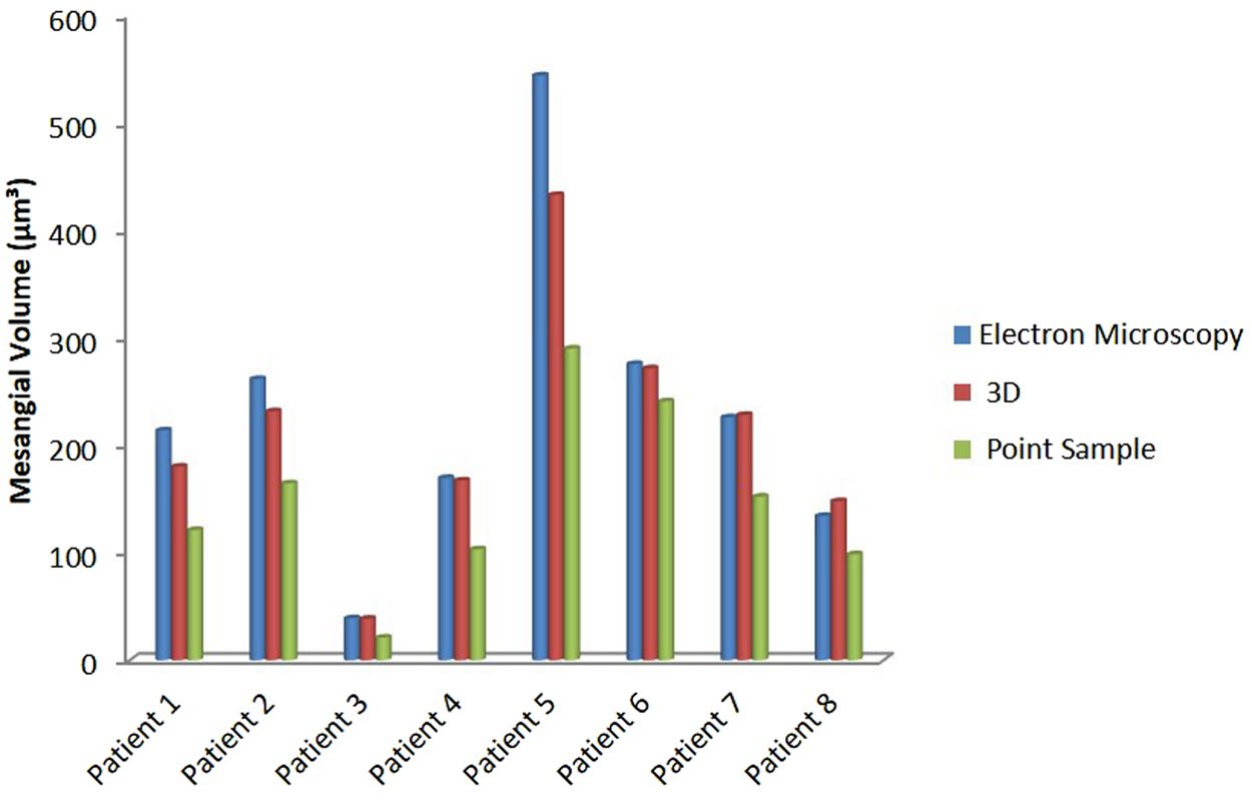
Comparison of three methods in measuring the mesangial volume. TEM (blue), 3D method (red) and PSI (green). Correlation coefficient between TEM/3D, TEM/PSI and 3D/PSI methods was 0.98, 0.93, and 0.96 respectively.

Mesangial volumes measured by the three methods are summarized in Fig. 3. Data from eight patients with measurements obtained using all three techniques were compared and statistically analysed.

Correlation coefficients between TEM/3D, TEM/PSI and 3D/PSI methods for mesangial volumes were 0.98, 0.93, and 0.96, respectively.

For an in-depth analysis of the result a multistep analysis was undertaken. We started by assessing if any difference existed between the three methods in general. Repeated-measures ANOVA test demonstrated a significant difference between the three groups in measurements. This was demonstrated by a statistically significant difference effect of measuring method on calculated mesangial volumes, F (2, 16) = 15.53, p = 0.0002.

To assess where this difference existed ANOVA post-estimation was used. It demonstrated that this was between PSI and TEM method with PSI method measurements being at a significantly smaller level of 30% (p = 0.0001). The two coefficients between 3D and TEM method measurements were not significantly different, at least at any significance level smaller than 3%. PSI measurements were also significantly smaller than 3D measurements by 15% (p = 0.0014).

In the eight patients that we measured mesangial volume, using the 3D method, we were able to show that the mesangial volume was greater in the macroalbuminuria group (n = 3) compared with normo/microalbuminuria patients (n = 5), 1117 µm³ ± 42 (Mean ± SD) vs 738 µm³ ± 48 (p < 0.05), respectively.

In all 22 patients, we were able to obtain mesangial and total glomerular area measurements using the “3D” technique. Using this method, we were also able to show that the ratio of mesangial to glomerular area was higher in macroalbuminuria (n = 8) compared with normo/microalbuminuria patients (n = 14), 0.76 ± 0.08 (Mean ± SD) vs 0.5 ± 0.13 (p < 0.01), respectively.

## Discussion

In the current study, we introduce a novel technique to measure mesangial volumes using three-dimensional (3D) imaging. Statistical analysis demonstrated a high correlation coefficient between the 3D and TEM method (gold standard method for this study) and no significant difference between the measurements (less than 3% variability) which is suggestive that 3D reconstruction is a reliable method to calculate mesangial volume. In comparison, the PSI method underestimated measurements compared to both methods, showing less accuracy while being more time consuming. Moreover, this study, using the novel reconstruction method confirmed the finding from our previous study which demonstrated that mesangial volume increases progressively from patients with type 2 diabetes and normo-, micro-, and macroalbuminuria^5^.

The pathophysiology of DKD is complex. Persistent hyperglycaemia and hypertension are risk factors, but it is unclear why some subsets of patients develop this complication whilst others do not. Furthermore, the structural changes which occur in the kidney that occur are known to differ between individuals with type 1 and type 2 diabetes^6^. While increasing albuminuria, especially macroalbuminuria, is a major factor in progression of diabetic kidney disease (DKD) and decline in glomerular filtration rate (GFR)^7^, a subset of patients with DKD show decline in renal function in the absence of elevated urinary albumin excretion rates (AER), so called “normoalbuminuric” decline in renal function^8,9^.

In patients with type 1 diabetes mellitus (T1DM) and reduced GFR, classic glomerular changes of DKD have been described regardless of albuminuria status^10^. By contrast, in patients with type 2 diabetes (T2DM) and DKD, regardless of the level of GFR or urinary AER, renal biopsy findings are generally accepted to be more heterogeneous than in type 1 diabetes^5^. Mesangial expansion, classically defined by mesangial fractional volume [Vv(mes/glom)] highly correlates with renal function changes in T1DM but changes in T2DM are less well characterised^10^. Moreover, to date, few studies have compared renal biopsy findings in subjects with type 2 diabetes, reduced GFR, and varying degrees of albuminuria, and as a result, the histological basis of normoalbuminuric DKD remains poorly understood.

In the current study, we have chosen to focus on mesangial volume as this is an important metric which correlates with the degree of renal function decline^8^ and also reflects the glomerular filtration surface area in diabetes^5^. The point-sampled intercept (PSI) method is the conventional method used to calculate this parameter. However, the PSI method is time consuming and is subject to underestimation^4,11^. The most attractive property of the 3D methods is that the methods can be applied to light-microscopy and is less subjective compared to the PSI.

Previous studies have measured the mesangial fractional volume [Vv(mes/glom)] using point counting methods in patients with and without diabetes^12–16^. Caramori *et al*.^12^ investigated the mesangial fraction volumes in control subjects and in patients with type 1 diabetes and variable levels of albuminuria. In the study by Caramori *et al*., mesangial fractional volume of 0.20 ± 0.03 (Mean ± SD) in the control group and 0.28 ± 0.07, 0.34 ± 0.09, 0.50 ± 0.12 (Mean ± SD) for patients with type 1 diabetes and normo, micro and macro albuminuria, respectively were reported^1^. Nasodini *et al*.^13^ examined the renal structure in patients with type 2 diabetes and demonstrated a mesangial fractional volume of 0.19 ± 0.03 (Mean ± SD) in the control group and 0.25 ± 0.05 and 0.3 ± 0.08 (Mean ± SD) in patients with micro and macro albuminuria, respectively. While our findings of 0.47 ± 0.15, 0.53 ± 0.09 and 0.76 ± 0.08 (Mean ± SD) in patients with normo, micro and macro albuminuria, respectively are higher values compared to the above studies, it should be noted that our biopsy specimens were all from patients with type 2 diabetes and with a mean estimated GFR (eGFR) of 42 ml/min/1.73 m^2^ which compares to the eGFR of the other studies which were 93 (ml/min/1.73 m^2^)^12^, 99 (ml/min/1.73 m^2^)^13^, 97 (ml/min/1.73 m^2^)^14^. Other factors apart from the type of diabetes and GFR, including duration of diabetes and glycaemic control, may impact the mesangial fractional volume and need to be accounted for when comparisons are made between studies that have documented mesangial fractional volumes in patients with diabetes.

In conclusion, we have shown for the first time that the 3D method is a promising method which could be more readily used in large scale future human and animal studies to describe the microanatomy of the kidney in DKD.

## Materials and Methods

### Sample preparation

This project was approved by Austin Health Human Research Ethics Committee and informed consent has been obtained for patients who had the biopsies taken for research purposes. The ethics committee approved waiver of consent for those who had renal biopsies for clinical reasons. All methods were performed in accordance with the relevant guidelines and regulations of our institution. Renal biopsies from 22 patients with type 2 diabetes and estimated glomerular filtration rate of <60 mL/min/1.73 m^2^ (Table 1) were obtained, fixed in formalin and embedded in paraffin for light microscopy. Patients were classified into normo-, micro-, or macroalbuminuria categories according to their 24 h urine albumin excretion rate. Tissue from renal biopsies was prepared for electron microscopy by fixation in 2.5% glutaraldehyde and resin embedded, and ultrathin sections were examined on a Jeol transmission electron microscope.

### Histology

Six ultra-thin serial horizontal (transverse) sections (1 μm) were cut from all blocks. Sections were stained with Periodic acid–Schiff–diastase (PAS) and Haematoxylin-Eosin (H&E) methods. Up to five glomerular units from each patient per section were photographed (x40) using an Olympus AX70 Provis microscope and a SPOT Flex 64 MP Colour FireWire Digital Camera, Diagnostic instruments Inc. The total number of glomeruli studied was 63 through a total of 320 serial sections.

### Quantitative analysis

Three methods were used to quantify mesangial volume (µm³), total mesangial area (µm²) and the ratio of mesangial/total area. These included a transmission electron microscopy (TEM) based method (using computer-assisted measurement), the PSI method and a novel new 3D imaging method. Due to the availability of sufficient samples for TEM, it was only possible to quantify mesangial volumes by all three methods in 8 patients. All patients (n = 22) had all metrics calculated using the 3D and PSI methods. A 3D representation was reconstructed from 63 glomeruli (320 serial sections) using “Reconstruct” (version 1.1.0.0) software (developed by J. C. Fiala and K. M. Harris at Boston University, MA, USA, 2007)^17^. Sections were aligned manually using four points of fixed structures in successive sections.

Mesangial area and volume was identified and quantified by the Reconstruct software based on staining differentiation resulting from PAS staining. EM assessment was performed using computer-assisted measurement through Digimizer (version 4.2.2) image analysis software (MedCalc Software, Ostend, Belgium). Mesangial area and estimated volume was obtained using an on-screen drawing tool to define the boundaries of each mesangial area. Transverse sections through the middle of representative glomeruli were chosen. For each subject, all identifiable mesangial area per glomerulus was measured. The mean mesangial area in a glomerulus per subject was then calculated.

The PSI method using Fiji (by Image J 1.51e, (NIH, Bethesda, MD, USA, 2016))^18^ software was also used to calculate fractional volumes of glomerular components by automated placement of grid lines on the section. This method utilizes Cavalieri’s principle^19^ and randomly placed lines that intersect the object at a specific point. This length is used to estimate a volume of the sampled objects.

### Statistical analysis

Eighteen individual data points from eight patients with measurements obtained using all three techniques were compared and statistically analysed. As a starting point correlation coefficient between results from the three methods was calculated with the TEM method being the gold-standard reference method.

To further investigate the difference between the methods and precisely establish the amplitude of any possible variation a multistep analysis was performed. As the first step, repeated-measures ANOVA test was used to test the equality of the measurement means from the TEM, PSI and 3D methods and investigate the degree of inter method variability. Subsequently, ANOVA postestimation test was used to specifically define where and to what degree differences were found by assessing the linear combinations of coefficients between TEM, PSI and 3D methods.

One-way ANOVA was used to test for differences among micro-, normo-, and macroalbuminuric groups for variables and T-tests were used to compare mesangial metrics between macro- and normo/microalbuminuric patients. Statistical significance was assigned at the *p* < 0.05 level. Microsoft Excel and STATA 15 were used for the statistical analyses.

## Data Availability

The associated data is attached and can be made available online.
